# Evaluating Potential Effects of Chamomile ( *Matricaria chamomilla* L.) in Polycystic Ovary Syndrome: A Systematic Review and Meta‐Analysis

**DOI:** 10.1002/fsn3.71600

**Published:** 2026-02-26

**Authors:** Zahra Firoozi, Roghayeh Molani‐Gol, Vahideh Ebrahimzadeh‐Attari, Aida Malek‐Mahdavi

**Affiliations:** ^1^ Student Research Committee Tabriz University of Medical Sciences Tabriz Iran; ^2^ Department of Biochemistry and Nutrition, Faculty of Nutrition and Food Sciences Tabriz University of Medical Sciences Tabriz Iran; ^3^ Tuberculosis and Lung Disease Research Center Tabriz University of Medical Sciences Tabriz Iran; ^4^ Connective Tissue Diseases Research Center Tabriz University of Medical Sciences Tabriz Iran

**Keywords:** Chamomile, clinical symptoms, *Matricaria chamomilla* L., Polycystic ovary syndrome, systematic review

## Abstract

The present study aimed to systematically assess the effects of Chamomile consumption on some biochemical parameters and clinical symptoms in patients with polycystic ovary syndrome (PCOS). The search was performed across the databases PubMed, Web of Science, Scopus, and Google Scholar using relevant keywords, with no language or date restrictions, up to the 4th of December 2024. Given the limited number of studies, results from both human and animal research were considered. The risk of bias for included studies was evaluated. For clinical studies, meta‐analyses were conducted using Stata, and a random‐effects model was used to estimate effect sizes. Four animal and four human studies were qualified for this review. Almost all animal studies indicated that Chamomile improved clinical, hormonal, and oxidative stress parameters in PCOS. Furthermore, almost all human studies have shown that Chamomile supplementation improves the clinical features of PCOS. However, the pooled analysis of two eligible human studies showed that there was no statistically significant effect of Chamomile supplementation on serum lipid profile including LDL‐C (ES = 1.73, 95% CI (−6.49, 9.94), *p* = 0.680), TG (ES = −7.23, 95% CI (−29.34, 14.89), *p* = 0.522), and HDL‐C (ES = −0.07, 95% CI (−5.85, 5.70), *p* = 0.980). Moreover, the pooled analysis of three eligible human studies demonstrated no statistically significant effect of Chamomile on serum testosterone levels (ES = 5.05, 95% CI = (−1.38, 11.49), *p* = 0.124). Overall, animal studies have demonstrated the potential benefits of Chamomile consumption in the treatment of PCOS, possibly through anti‐androgenic, anti‐inflammatory, analgesic, and antioxidant effects. In contrast, human studies yielded heterogeneous findings, with improvements in clinical features but no significant impact on serum testosterone or lipid profile parameters. Therefore, further well‐designed clinical trials are needed to make more definitive decisions.

## Graphical abstract

Chamomile contains different bioactive agents such as flavonoids and phytosterols with potential anti‐inflammatory, antioxidant, analgesic, antispasmodic, and antitumor characteristics. According to our findings, animal studies have demonstrated the potential benefits of Chamomile consumption in the treatment of PCOS. In contrast, human studies yielded heterogeneous findings, with improvements in clinical features but no significant impact on serum testosterone or lipid profile parameters.

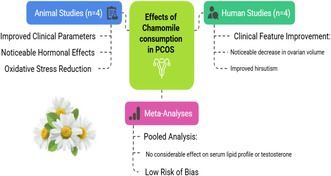

## 1 Introduction

Polycystic ovarian syndrome (PCOS) is a multifaceted endocrine disease characterized by the presence of ovarian cysts, anovulation, and significant hormonal imbalances. The disturbances in the reproductive hormones disrupt the regular menstrual cycle, which can contribute to oligomenorrhea, amenorrhea, hirsutism, acne, and insulin resistance (Bulsara et al. 2021). On the basis of a recent report by the World Health Organization, about 116 million (3.4%) females of childbearing age are diagnosed with PCOS around the world. The risk factors that result in the expansion of PCOS include genetics, neuroendocrine, lifestyle/environment, and obesity (Bulsara et al. 2021; Singh et al. 2023).

Given the complicated etiology of PCOS, treatment is rarely monotherapeutic and instead is personalized on the basis of the individual's clinical signs. Various pharmacological interventions, including oral contraceptives, anti‐androgens, insulin sensitizers, ovulation‐inducing agents, and anti‐obesity drugs, are used to alleviate symptoms (Singh et al. 2023). Nevertheless, long‐term consumption of medications can initiate significant complications like menstrual abnormalities, gastrointestinal complications, and weight gain. Non‐pharmacological treatments, such as diet therapy and lifestyle modifications (e.g., regular physical activity and yoga), play important roles in the prevention and management of PCOS. Adopting a balanced diet rich in whole grains, proteins, fruits, vegetables, and healthy fats helps to modulate blood sugar concentrations and improve insulin sensitivity. In addition, the use of nutritional supplements such as vitamins (B9, B12, D, E, and K), minerals (calcium, zinc, selenium, and chrome), and other supplements (flavonoids, omega‐3 fatty acids, coenzyme Q10, probiotics, melatonin, etc.) may have some beneficial effects (Maan et al. 2025; Zhao et al. 2022, 2023).

Recently, medicinal plants have been considered promising agents for PCOS treatment, with fewer side effects than conventional therapies (Lakshmi et al. 2023). Among them, Chamomile has significant research value because of its wide array of compounds, diverse pharmacological effects, broad geographical distribution, widespread use, and general safety. Chamomile (*Matricaria chamomilla L*.) is related to the *Asteraceae* family, which grows in different regions of the world. The most significant part of Chamomile utilized for therapeutic objectives is its flower, which contains different bioactive agents such as terpenoids, flavonoids, tannins, phytosterols like coumarins, organic acids, and mucilages (Chauhan and Aishwarya 2018). This plant has been traditionally used to treat numerous diseases, including gastrointestinal disorders, neuropsychiatric issues, common cold and respiratory problems, and skin, eye, and mouth diseases (El Mihyaoui et al. 2022). Chamomile has been reported to exhibit various pharmacologic effects, including anti‐inflammatory, antioxidant, analgesic, anti‐microbial, anti‐allergic, anti‐spasmodic, anti‐tumor, anti‐hypertensive, hypolipidemic, anti‐diabetic, anti‐depressant, neuroprotective, and hepatoprotective (Sah et al. 2022). Furthermore, Chamomile has an anti‐estrogenic effect and can regulate menstrual cycles (El‐Halawany et al. 2011). According to a recent systematic review, Chamomile is generally safe when used in controlled dosages, with self‐limiting minor adverse events. However, allergic reactions should be taken into consideration. (Ostovar et al. 2025).

There are recent reports on its protective effects in PCOS (Afiat et al. 2024, 2022; Dadmehr et al. 2023; Heidary et al. 2018; Shamsi et al. 2023; Alahmadi et al. 2021, 2020; Zafari Zangeneh et al. 2010). According to them, considerable attenuation of hirsutism (Afiat et al. 2024, 2022), increased numbers of dominant follicles (Afiat et al. 2024; Zafari Zangeneh et al. 2010), and lower levels of total testosterone (Heidary et al. 2018; Alahmadi et al. 2021) were demonstrated in the Chamomile group. It seems that Chamomile may reduce estrogen and testosterone by inhibiting aromatase and Cytochrome P450 enzymes, and by exerting negative feedback on luteinizing hormone (LH). Chamomile polyphenols may reduce inflammation associated with hyperandrogenism by inhibiting cyclooxygenase‐2 (COX‐2) (Dadmehr et al. 2023; Heidary et al. 2018). To the best of our knowledge, no prior systematic review or meta‐analysis has comprehensively assessed preclinical and/or clinical evidence on the effects of Chamomile on PCOS. Therefore, to fill this knowledge gap, the present study aimed to systematically evaluate results of both animal and human studies investigating the potential clinical, hormonal, metabolic, and oxidative stress–related effects of Chamomile supplementation in patients with PCOS.

## 2 Methods

### 2.1 Study Protocol and Strategy of Search

This study was in agreement with the Preferred Reporting Items for Systematic Reviews and Meta‐Analyses (PRISMA) instructions (Table S1). The study protocol was registered with the International Prospective Register of Systematic Reviews (PROSPERO) under registration number CRD42024562512. A literature search was conducted using electronic databases, including Web of Science, PubMed, Scopus, and Google Scholar, up to the 4th of December 2024. The following MESH and non‐MESH words were applied in the title, abstract, and keywords: “Chamomile”, “Chamomile Oil”, “Oil, Chamomile”, “Polycystic Ovary Syndrome”, “polycystic ovarian syndrome”, “PCOS”, without imposing any restrictions on language, publication date, or an intended variable. The search strategy is demonstrated in Table S2. Two researchers (ZF, AMM) separately performed the search and screening activities. Duplicates were recognized and eliminated. The references of relevant papers were also assessed to uncover related studies. The two authors reached consensus on article selection, and any potential disagreements were resolved by the third investigator (VEA).

### 2.2 Inclusion and Exclusion Criteria

The population, intervention, comparison, and outcome (PICO) criteria of the current review are shown in Table 1. Investigations that met the following criteria were included: assessing the effect of Chamomile intake on PCOS compared to the control group; published in any language; and accessible in full text. Exclusion criteria were: review papers; book chapters; gray literature (theses, conference abstracts, and patents); evaluating the effects of Chamomile in other diseases; assessing the effect of Chamomile together with other compounds, wherein the influence of Chamomile could not be demonstrated alone.

**TABLE 1:** The PICOS criteria for selecting the studies.

Criteria	Description
Population	PCOS patients
Intervention	Chamomile
Comparison	Control group (placebo or no intervention)
Outcome	Change in clinical and biochemical variables of PCOS patients
Study design	In vitro studies, experimental animal and human studies

Abbreviation: PCOS, polycystic ovarian syndrome.

### 2.3 Data Extraction

Two authors (ZF and AMM) performed the data extraction. Data of the first author's surname, issue year, sample details, type and dose of Chamomile administered, period of intervention, and reported outcomes were extracted from the selected studies. Moreover, the mean and standard deviation (SD) of the randomized controlled trials (RCTs) were extracted for meta‐analysis.

### 2.4 Risk of Bias Evaluation

The risk of bias (RoB) in the included RCTs and animal research was evaluated by two authors using the Cochrane Collaboration's tool and the Office of Health Assessment and Translation (OHAT) tool, respectively. The Cochrane Collaboration's tool includes six bias domains, and each domain was judged to have a low, unclear, or high risk. About the OHAT tool, the response options per question are low risk of bias (+ +), definitely high risk of bias (− −), probably low risk of bias (+), or probably high risk of bias (−). Finally, each question gave a numerical value from −2 to +2, and studies were tiered on the basis of the obtained averages. Tier 1 investigations demonstrated a low risk of bias; Tier 2 investigations, a moderate risk; and Tier 3 investigations, a high risk.

### 2.5 Statistical Analysis

We performed the meta‐analysis only for human studies, including 232 participants, because a reliable meta‐analysis was not possible for animal studies because of limited numbers and comparable outcomes. The mean change in each group was calculated as the difference between the mean value at the end of the follow‐up and the baseline. To compute the SD changes, the following formula was applied: SD = square root [(SD pre‐treatment)^2^ (SD post‐treatment)^2^–(2R × SD pre‐treatment × SD post‐treatment)]. The correlation coefficient (*R*‐value) was assumed to be 0.8, consistent with previous meta‐analyses (Borenstein et al. 2021). This assumption may introduce uncertainty and should be considered when interpreting the results. The random‐effect model was used to pool the included studies' results and to report weighted mean differences (WMD) and 95% confidence intervals (CI). The heterogeneity of included investigations was evaluated using the Cochrane's Q test (*p*‐value < 0.1) and the I2 statistic (I2 > 50%, indicating considerable heterogeneity). Because of the small number of RCTs, we are unable to conduct subgroup and sensitivity analyses. The meta‐analysis was performed using Stata 17.0 (StataCorp, College Station, TX), and *p* < 0.05 was considered statistically significant.

## 3 Results

### 3.1 Study Selection

**FIGURE 1:**
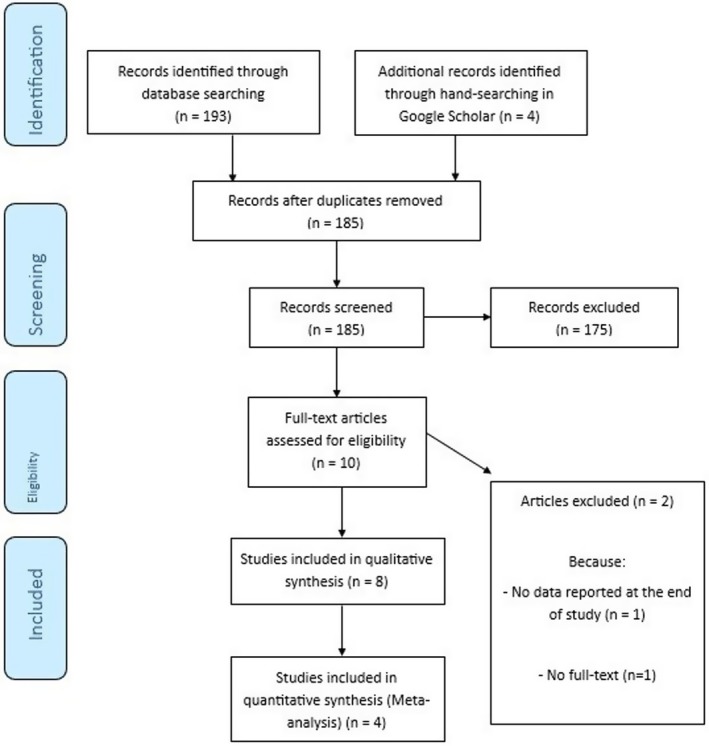
PRISMA flow diagram of the literature search and study selection process.

As shown in Figure 1, a total of 197 articles were identified through the electronic database search (PubMed = 8, Scopus = 180, Web of Science = 5, Google Scholar = 4). After removing duplicates (*n* = 12), 185 studies remained for screening titles and abstracts. Then, 175 studies were excluded as irrelevant to the research question. Two further articles were also excluded because of no data being reported at the end of the study (*n* = 1) and the lack of full‐text availability (*n* = 1). Finally, eight eligible studies (four animal and four human models) were included. No relevant in vitro research was noticed. Tables 2 and 3 provide a summary of animal studies and clinical studies, respectively.

**TABLE 2:** Summary of animal studies (*n* = 4).

First author, year	Country	Species	Sample size	Intervention	Dose of intervention	Route of intervention	Duration of intervention	Outcomes
Shamsi et al. 2023	Iran	DHEA‐induced PCOS BALB/C mice	*N* = 30	Chamomile extract	500 mg/kg/day	Intraperitoneal injection	21 days	Decrease in the number of cystic follicles compared to the PCOS control group. Increase in antral and corpus luteum follicles compared to the PCOS control group. No change in the number of preantral follicles compared to the PCOS control group Increase in serum FRAP levels compared to the PCOS control group No change in serum glucose levels compared to the PCOS control group
Alahmadi et al. 2021	Saudi Arabia	PCOS‐induced Wistar rats	*N* = 24	Chamomile flower extract	75 mg/kg/day	Oral	30 days	Decrease in serum testosterone compared to the PCOS control group No change in body weight mass compared to the PCOS control group Decrease in serum lipid peroxide product Increase in serum glutathione, catalase, and superoxide dismutase compared to the PCOS control group
Alahmadi et al. 2020	Saudi Arabia	Estradiol valerate‐induced PCOS Wistar rats	*N* = 24	Chamomile flower extract	75 mg/kg/day	Oral	30 days	No change in body weight mass compared to the PCOS control group Decrease in serum estrogen compared to the PCOS control group Decrease in serum MDA level compared to the PCOS control group Increase in levels of serum glutathione, glutathione peroxidase, and catalase compared to the PCOS control group No change in serum superoxide dismutase compared to the PCOS control group
Zafari Zangeneh et al. 2010	Iran	Virgin adult Wistar PCOS rats	*N* = 30	Chamomile alcoholic extract	25, 50, 75 mg/kg/day	Intraperitoneal injection	10 days	Recovery in macroscopic and microscopic morphological examination in the ovarian and uterine tissues only at the dose 50 mg/kg/day compared to the PCOS control group Increase in the number of dominant follicles compared to the PCOS control group Better endometrial tissue arrangements compared to the PCOS control group Decrease in serum levels of estradiol, gonadotropins, LH, and FSH compared to the PCOS control group

Abbreviations: DHEA, dehydroepiandrosterone; FRAP, serum antioxidant capacity; FSH, follicle‐stimulating hormone; LH, luteinizing hormone; MDA, malondialdehyde; PCOS, polycystic ovarian syndrome.

**TABLE 3:** Summary of clinical studies (*n* = 4).

First author, year	Country	Population	Sample size	Intervention	Dose of intervention	Route of intervention	Duration of intervention	Outcomes
Afiat et al. 2024	Iran	PCOS patients	*N* = 70	Chamomile capsules	1000 mg/day	Oral	3 months	Significantly lower ovarian volume on day 12 of the third cycle compared to the PCOS control group Significantly higher number of dominant follicles in the third cycle compared to the PCOS control group Improvement in hirsutism compared to the PCOS control group No significant change in serum testosterone levels compared to the PCOS control group Borderline significant decrease in oligomenorrhea compared to the PCOS control group
Dadmehr et al. 2023	Iran	PCOS patients	*N* = 12	Chamomile oil	1 mL	Topical	10 min once every night for three consecutive cycle periods	Decrease in pain intensity
Afiat et al. 2022	Iran	PCOS patients	*N* = 70	Chamomile capsules	1000 mg/day	Oral	3 months	Significant decrease in hirsutism compared to the PCOS control group No significant change in oligomenorrhea compared to the PCOS control group No significant change in FBS, HDL‐C, cholesterol, triglyceride, testosterone, and LDL‐C compared to the PCOS control group
Heidary et al. 2018	Iran	PCOS patients	*N* = 80	Chamomile capsules	1110 mg/day	Oral	3 months	Significant decrease in serum testosterone compared to the PCOS control group No significant change in serum LDL‐C, HDL‐C, triglycerides, DHEA‐sulfate, and the ratio of LH/FSH compared to the PCOS control group

Abbreviations: DHEA: dehydroepiandrosterone; FSH: follicle‐stimulating hormone; HDL‐C: high‐density lipoprotein cholesterol; LDL‐C: low‐density lipoprotein cholesterol; LH, luteinizing hormone; PCOS, polycystic ovarian syndrome.

### 3.2 Study Characteristics

As shown in Table 2, all the animal studies were conducted in Saudi Arabia or Iran and published between 2010 and 2023. The animal studies involved rats or mice, and the number of animals ranged from 24 to 30. Moreover, the treatment dose and duration were between 25 to 500 mg/kg/day and 10–30 days, respectively.

As shown in Table 3, all included RCTs were conducted in Iran and published between 2018 and 2024. The intervention dosage ranged from 1000 to 1110 mg/day, and the supplementation duration in all included studies was approximately 3 months. The study design in all studies was parallel. The eligible trials included 12 to 80 PCOS patients, and a total of 232 subjects participated in the RCTs. It should be noted that the limited number of eligible studies (4 RCTs and 4 animal studies) reduces the study's power, and this should be considered a limitation for the present study.

### 3.3 Animal Study Findings

The effects of Chamomile on PCOS clinical parameters were examined in two of four animal studies (Shamsi et al. 2023; Zafari Zangeneh et al. 2010). Both studies showed that intraperitoneal injection of Chamomile extract led to significant signs of recovery in uterine and ovarian tissues, including decreased numbers of cystic follicles, increased numbers of antral and corpus luteum follicles, and improved endometrial tissue organization in PCOS‐induced animals compared with the PCOS control group (Table 2).

The effects of Chamomile on hormonal factors were examined in three of four animal studies (Alahmadi et al. 2021, 2020; Zafari Zangeneh et al. 2010). According to these studies, serum levels of testosterone, estrogen, estradiol, gonadotropins, LH, and follicle‐stimulating hormone (FSH) were significantly reduced by Chamomile extract in PCOS‐induced rats compared to the control group (Table 2).

The effects of Chamomile on PCOS body weight were examined in two of four animal studies (Alahmadi et al. 2021, 2020). On the basis of these studies, supplementation with Chamomile flower extract did not significantly affect body weight in PCOS‐induced Wistar rats vs. the control group (Table 2). Furthermore, the effects of Chamomile on metabolic factors were examined in one of four animal studies (Shamsi et al. 2023), which indicated that intraperitoneal injection of Chamomile extract did not significantly alter blood sugar levels in DHEA‐induced PCOS mice compared to the PCOS group (Table 2).

The effects of Chamomile on oxidative stress factors were examined in three of four animal studies (Shamsi et al. 2023; Alahmadi et al. 2021, 2020). According to these studies, Chamomile extract significantly increased serum antioxidant capacity (FRAP), GSH, CAT, superoxide dismutase (SOD), and glutathione peroxidase (GPx) levels in PCOS‐induced animals compared with the PCOS control group (Table 2). In addition, serum lipid peroxide product levels were significantly lower in the Chamomile extract group than in the control group.

### 3.4 Human Study Findings

The effects of Chamomile on PCOS clinical parameters were examined in three of four human studies (Afiat et al. 2024, 2022; Dadmehr et al. 2023). According to Afiat et al.'s studies (Afiat et al. 2024, 2022), Chamomile supplementation led to a significant decrease in ovarian volume on day 12 of the third menstrual cycle, a higher number of dominant follicles, and improved hirsutism without affecting oligomenorrhea compared with the placebo group (Table 3). Furthermore, in the study by Dadmehr et al. (2023), applying Chamomile oil topically for three consecutive cycles significantly reduced the intensity of menstrual pain in patients with confirmed PCOS‐related dysmenorrhea (Table 3).

The effects of Chamomile on hormonal factors were examined in three of four human studies (Afiat et al. 2024, 2022; Heidary et al. 2018). Two studies by Afiat et al. (Afiat et al. 2024, 2022) found that after Chamomile supplementation vs. placebo, serum testosterone levels did not differ considerably between the two groups (Table 3). Additionally, in a randomized clinical trial by Heidary et al. (Heidary et al. 2018), serum testosterone levels were significantly lower in PCOS women following the Chamomile intervention. In contrast, there was no discernible difference between the experimental and control groups' levels of the hormone DHEA‐sulfate or the ratio of LH/FSH (Table 3).

**FIGURE 2:**
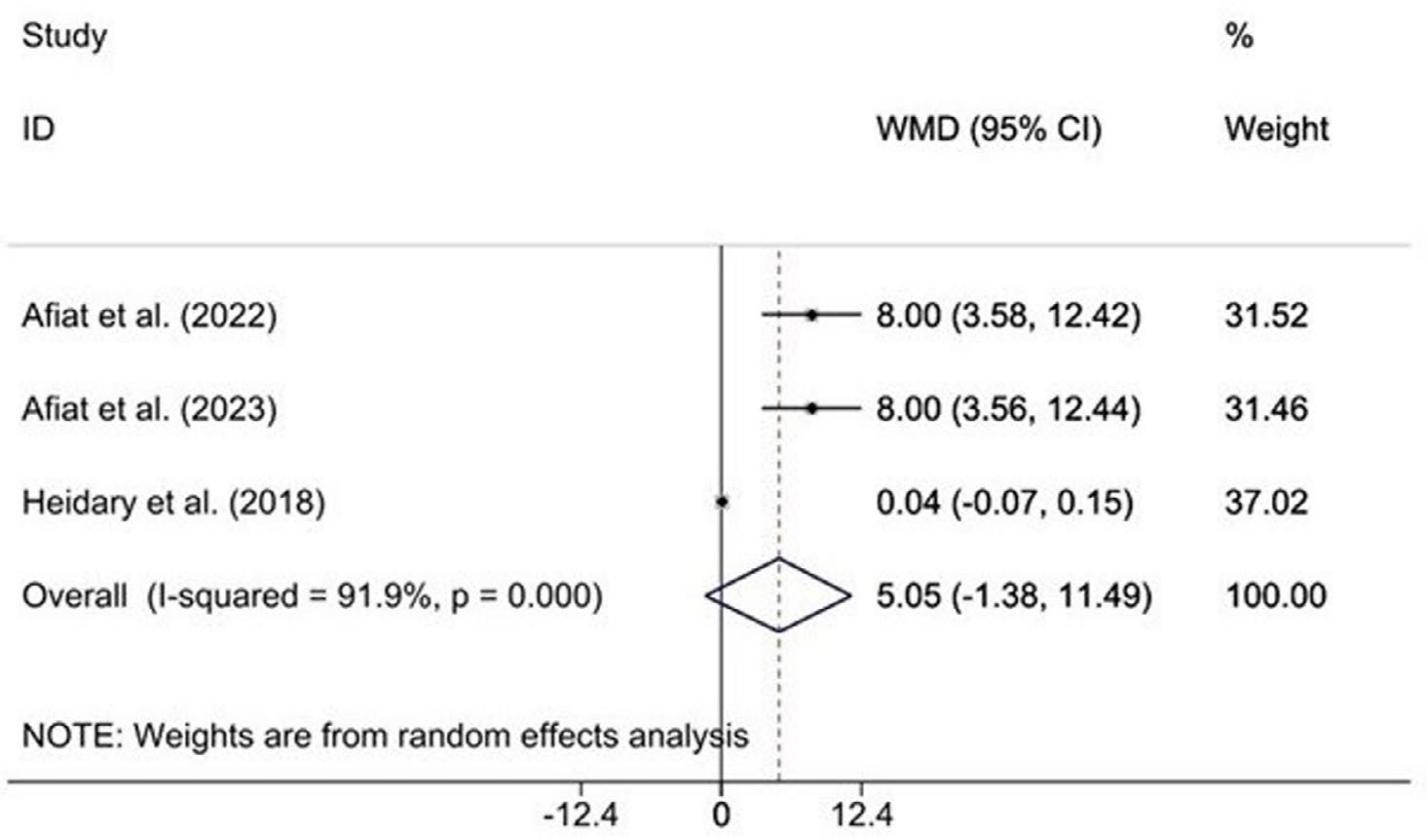
Forest plot detailing mean difference (MD) and 95% confidence intervals (CIs) for the effects of Chamomile supplementation on testosterone levels in patients with polycystic ovary syndrome.

Meta‐analysis results of the effects of Chamomile supplementation on testosterone levels are presented in Figure 2. As indicated in this forest‐plot, three randomized controlled clinical trials (RCTs) comprising 220 participants examined the effect of Chamomile consumption on testosterone levels, and pooled ESs from these studies revealed no significant change in testosterone concentration (ES = 5.05, 95% CI (−1.38, 11.49), *p* = 0.124) (Figure 2). The results showed high heterogeneity among the included clinical trials (I^2^ = 91.9%, *p* = 0.000). Considerable heterogeneity indicates substantial variability in study outcomes. Because of the limited number of included studies, we could not conduct subgroup or sensitivity analyses.

The effects of Chamomile on metabolic factors were examined in two of four human studies (Afiat et al. 2022; Heidary et al. 2018); however, no human study evaluated the effects of Chamomile on body weight. In both studies, there was no noticeable difference in fasting blood sugar (FBS), high‐density lipoprotein cholesterol (HDL‐C), low‐density lipoprotein cholesterol (LDL‐C), total cholesterol, and triglycerides (TG) between the Chamomile and placebo PCOS groups (Table 3).

**FIGURE 3:**
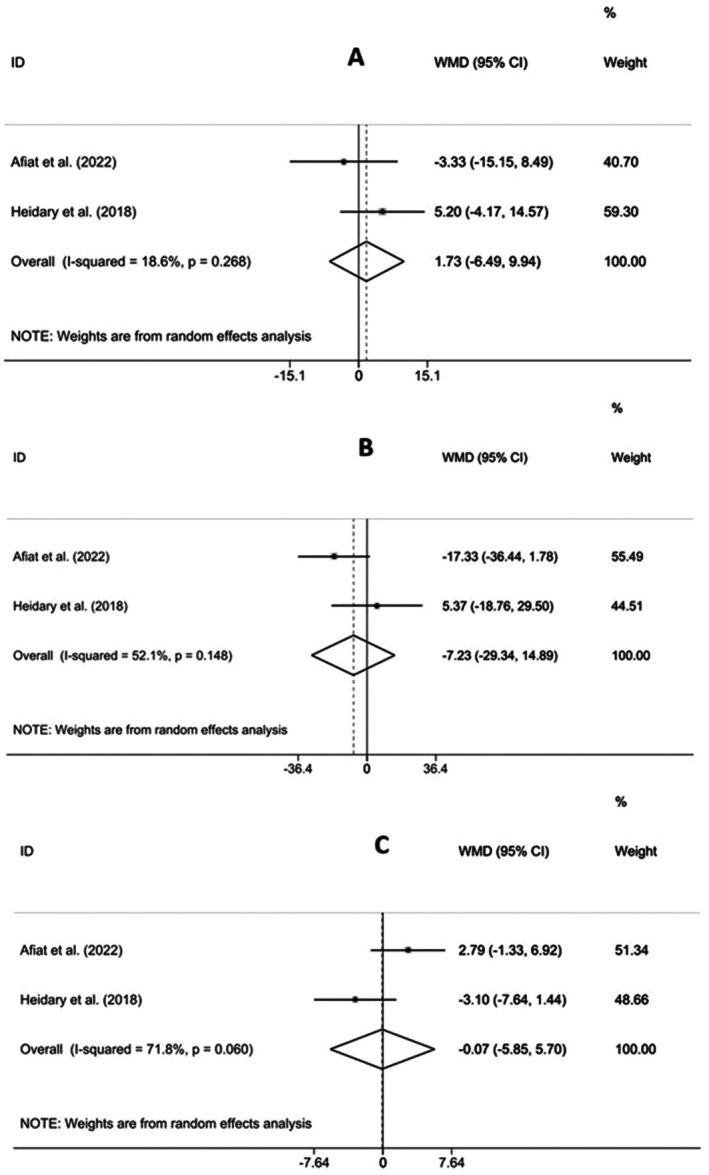
Forest plot detailing mean difference (MD) and 95% confidence intervals (CIs) for the effects of Chamomile supplementation on low‐density lipoprotein cholesterol (A), triglyceride (B), and high‐density lipoprotein cholesterol (C) in patients with polycystic ovary syndrome.

Meta‐analysis results of the effects of Chamomile supplementation on lipid profiles are presented in Figure 3A–C. As shown in these forest‐plots, combining the findings of two included studies involving 150 participants that evaluated the effects of Chamomile consumption on lipid profiles demonstrated that Chamomile supplementation had no statistically significant effects on improving serum concentrations of LDL‐C (ES = 1.73, 95% CI (−6.49, 9.94), *p* = 0.680) (Figure 3A), TG (ES = −7.23, 95% CI (−29.34, 14.89), *p* = 0.522) (Figure 3B), and HDL‐C (ES = −0.07, 95% CI (−5.85, 5.70), *p* = 0.980) (Figure 3C). There was low heterogeneity among the research for LDL‐C (I^2^ = 18.6%, *p* = 0.268), HDL‐C (I^2^ = 71.8%, *p* = 0.060), and TG (I^2^ = 52.1%, *p* = 0.148). Because of the small number of included investigations, we couldn't conduct the subgroup and sensitivity analyses.

### 3.5 Risk of Bias Evaluation

On the basis of the quality evaluation of the included RCTs using the Cochrane Collaboration scale (Table S3), all studies demonstrated a low risk of bias across key domains, including random sequence generation, allocation concealment, and outcome assessment, and were of low risk of bias. Moreover, three animal studies have a low risk of bias, and one has a moderate risk of bias according to the OHAT tool (Table S4).

## 4 Discussion

To the authors' knowledge, the current systematic review and meta‐analysis is the first study to investigate the effect of Chamomile supplementation on various variables in PCOS. Almost all animal studies reported that Chamomile improved clinical, hormonal, and oxidative stress parameters in PCOS (Zafari Zangeneh et al. 2010; Alahmadi et al. 2021, 2020; Shamsi et al. 2023). Furthermore, nearly all human studies have shown that Chamomile supplementation improves the clinical features of PCOS (Afiat et al. 2024, 2022; Heidary et al. 2018; Dadmehr et al. 2023). The pooled analysis of two eligible RCTs indicated that Chamomile consumption had no significant effect on serum LDL‐C, TG, and HDL‐C levels (Afiat et al. 2022; Heidary et al. 2018). Moreover, the pooled analysis of three eligible RCTs showed that Chamomile consumption had no statistically significant effect on serum testosterone levels (Afiat et al. 2024, 2022; Heidary et al. 2018). The substantial heterogeneity observed in the pooled analysis may be attributable to several factors, including differences in Chamomile formulations, dosages, intervention durations, participants' baseline hormonal status, and study designs. Additionally, variations in bioavailability and metabolic responses among individuals with PCOS may have contributed to inconsistent findings.

The discrepancy between Chamomile's effects on hormonal parameters in clinical and animal investigations may be due to differences in study models, preparations, administration routes, doses, bioavailability, and treatment duration. Furthermore, non‐significant results in the included RCTs may be due to variation in intervention dose and duration, as well as differences in participants' metabolic properties. However, this study was consistent with a previous systematic review of patients with primary dysmenorrhea, which found that Chamomile was effective in reducing pain severity (Niazi and Moradi 2021).

Contrary to our findings on the lipid profile of PCOS patients, an RCT by Rafraf et al. (2015) in type 2 diabetic subjects reported a statistically significant reduction in serum total cholesterol, LDL‐C, and TG concentrations following Chamomile tea consumption. However, our study was consistent with theirs, as there was no statistically significant change in HDL‐C levels following Chamomile consumption (Rafraf et al. 2015). Since no similar systematic review or meta‐analysis had investigated the effects of Chamomile on PCOS and related parameters, we could not compare our results.

The selected studies regarding Chamomile supplementation on PCOS demonstrate multifaceted mechanisms of action, primarily involving anti‐androgenic, anti‐inflammatory, analgesic, and antioxidant effects. Hyperandrogenism, driven by ovarian androgen secretion, is the main characteristic of PCOS and plays a role in initiating metabolic disturbances, inflammation, and oxidative stress (Bulsara et al. 2021).

Chamomile extract contains phytoestrogens, such as coumarins, which may reduce estrogen production (Brueggemeier et al. 2001). Phytoestrogens and coumarin exert negative feedback on LH secretion and can also inhibit the anti‐androgenic receptor complex, thereby decreasing testosterone secretion (Karampoor et al. 2014). Phytoestrogens inhibit the function of cytochrome P450 enzymes, thereby preventing cholesterol from being converted to pregnenolone and reducing the production of steroids, including testosterone (Ronis 2016). Chamomile also exhibits progestogenic properties that are effective in PCOS treatment (Mirzakhani and Hosseini 2017).

Moreover, increasing evidence indicates that long‐term inflammation plays a critical role in PCOS development and follicular dysplasia pathogenesis (Aboeldalyl et al. 2021). Long‐term chronic inflammation contributes to mitochondrial dysfunction, influencing the energy supply and thus modifying ovarian activity, ovulation, fertilization, and insemination in PCOS subjects (Repaci et al. 2011). A significant relationship exists between circulating inflammatory biomarkers, oxidative stress, and androgen concentrations (Zhao et al. 2015). Reports have affirmed that PCOS individuals are in a chronic condition of oxidative stress imbalance, and oxidative stress has become a primary parameter in the pathogenesis of PCOS (Murri et al. 2013). In addition, oxidative stress and inflammatory markers are linked with insulin resistance (González et al. 2006).

**FIGURE 4:**
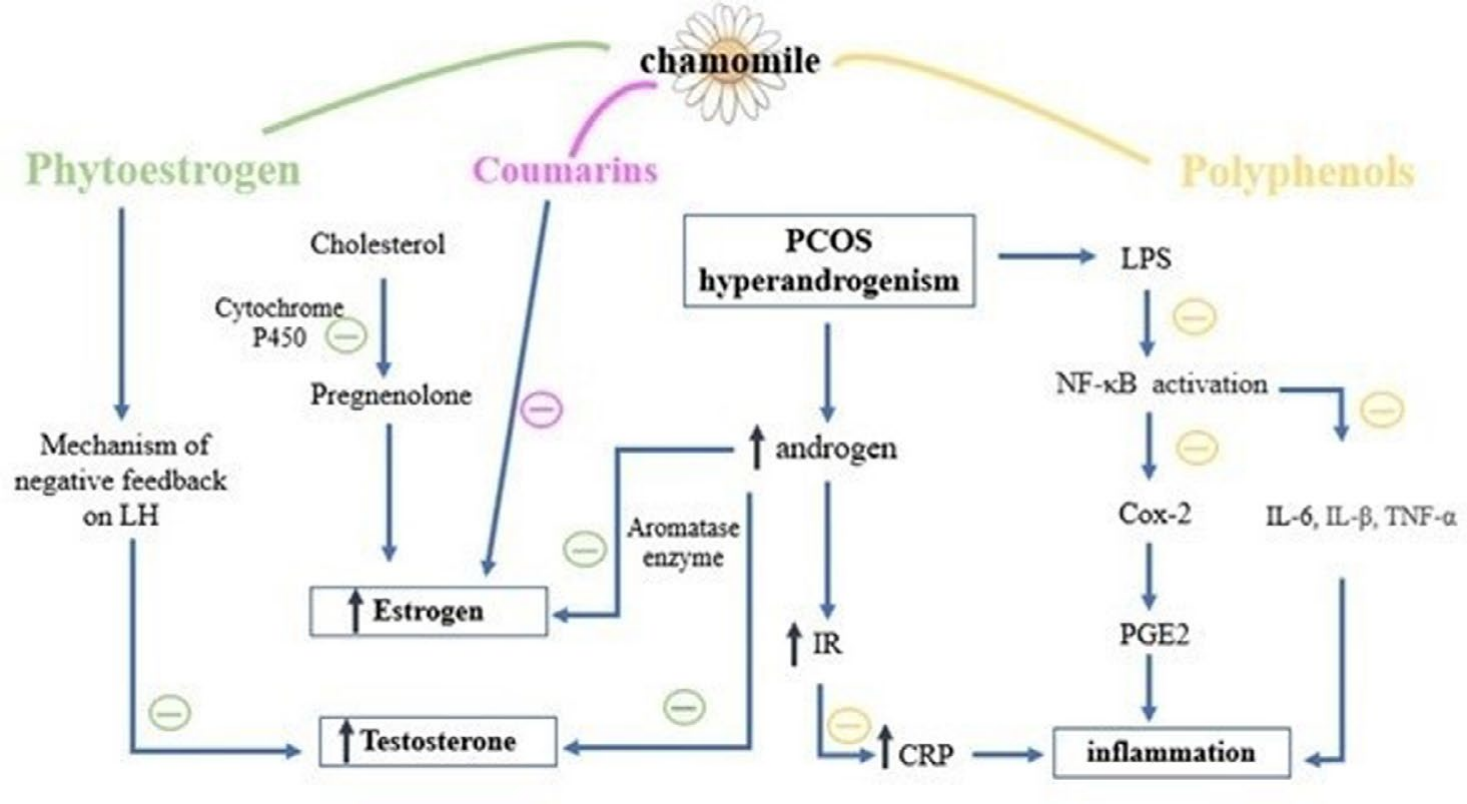
The mechanism of action of Chamomile in polycystic ovary syndrome.

Chamomile has analgesic and anti‐inflammatory activities and can diminish the production of prostaglandins and leukotrienes in the endometrium, as one of the leading causes of pain in dysmenorrhea (Niazi and Moradi 2021). The flavonoids in Chamomile have a strong suppressing effect on the prostaglandin E2 level and have an anti‐inflammatory effect by affecting the cyclooxygenase‐2 (COX‐2) pathway. These mechanisms can justify the anti‐inflammatory and analgesic effects of Chamomile (Hashempur et al. 2017). Figure 4 summarizes the mechanism of action of Chamomile in polycystic ovary syndrome. It should be noted that many of the proposed mechanisms described above are primarily derived from animal studies or indirect biochemical evidence.

There are some limitations for this review. The first limitation of the present study was the limited number of RCTs, which prevented us from conducting the subgroup and sensitivity analyses. Furthermore, the small number of included studies is another potential limitation, which may affect the generalizability and robustness of the findings. Another important limitation is that all included RCTs were conducted in the Iranian population, which may limit the generalizability of the findings. Therefore, the reports of our meta‐analyses should be interpreted with caution, and additional clinical trials in different ethnicities are necessary to evaluate the effect of Chamomile supplementation on various parameters of subjects with PCOS.

Generally, animal and clinical studies have shown that Chamomile supplementation has advantages in improving clinical manifestations in women with PCOS. Although Chamomile supplementation had beneficial effects on hormone levels in animal models of PCOS, the current meta‐analysis did not demonstrate any significant effect of Chamomile on serum testosterone levels and lipid profile in PCOS women. Therefore, clinical efficacy remains inconclusive, and further well‐designed clinical trials in diverse populations—incorporating standardized Chamomile extract formulations, extended intervention durations, and larger sample sizes—are needed to improve external validity and support more comprehensive definitive conclusions.

## Author Contributions

**Aida Malek‐Mahdavi:** conceptualization, writing – review and editing, data curation, methodology. **Vahideh Ebrahimzadeh‐Attari:** data curation, supervision, writing – review and editing, methodology, writing – original draft, project administration.

## Conflicts of Interest

The authors declare no conflicts of interest.

## Supporting information


**Table S1:** PRISMA checklist of study.
**Table S2:**. Method of the database search strategy using PubMed, Scopus, and Web of Science.
**Table S3:**. Cochrane Collaboration scale for assessment of quality of the included randomized controlled trials (RCTs).
**Table S4:**. The risk of bias assessment and tier classifications of animal studies (OHAT tool).

## Data Availability

The data that support the findings of this study are available in the Tables S1–S4 and Figures 2 and 3.
