# Supplementary material for: Evaluating Potential Effects of Chamomile ( Matricaria chamomilla L.) in Polycystic Ovary Syndrome: A Systematic Review and Meta‐Analysis
Source: Food Sci Nutr. 2026 Feb 26;14(3):e71600. doi: 10.1002/fsn3.71600 (PMC12945713; doi:10.1002/fsn3.71600)
Supplement: Supplementary file 1 — Table S1: PRISMA checklist of study. Table S2:. Method of the database search strategy using PubMed, Scopus, and Web of Science. Table S3:. Cochrane Collaboration scale for assessment of quality of the included randomized controlled trials (RCTs). Table S4:. The risk of bias assessment and tier classifications of animal studies (OHAT tool). [file FSN3-14-e71600-s001.docx]

**Supplementary Table 1.** PRISMA checklist of study

| **Section/topic** | **#** | **Checklist item** | **Reported on page #** |
| --- | --- | --- | --- |
| **TITLE** | | |  |
| Title | 1 | Identify the report as a systematic review, meta-analysis, or both. | 1 |
| **ABSTRACT** | | |  |
| Structured summary | 2 | Provide a structured summary including, as applicable: background; objectives; data sources; study eligibility criteria, participants, and interventions; study appraisal and synthesis methods; results; limitations; conclusions and implications of key findings; systematic review registration number. | 2 |
| **INTRODUCTION** | | |  |
| Rationale | 3 | Describe the rationale for the review in the context of what is already known. | 3 |
| Objectives | 4 | Provide an explicit statement of questions being addressed with reference to participants, interventions, comparisons, outcomes, and study design (PICOS). | 4 |
| **METHODS** | | |  |
| Protocol and registration | 5 | Indicate if a review protocol exists, if and where it can be accessed (e.g., Web address), and, if available, provide registration information including registration number. | 4 |
| Eligibility criteria | 6 | Specify study characteristics (e.g., PICOS, length of follow-up) and report characteristics (e.g., years considered, language, publication status) used as criteria for eligibility, giving rationale. | 5 |
| Information sources | 7 | Describe all information sources (e.g., databases with dates of coverage, contact with study authors to identify additional studies) in the search and date last searched. | 4 |
| Search | 8 | Present full electronic search strategy for at least one database, including any limits used, such that it could be repeated. | Supplementary Table 2 |
| Study selection | 9 | State the process for selecting studies (i.e., screening, eligibility, included in systematic review, and, if applicable, included in the meta-analysis). | 5 |
| Data collection process | 10 | Describe method of data extraction from reports (e.g., piloted forms, independently, in duplicate) and any processes for obtaining and confirming data from investigators. | 5 |
| Data items | 11 | List and define all variables for which data were sought (e.g., PICOS, funding sources) and any assumptions and simplifications made. | 4, 5 |
| Risk of bias in individual studies | 12 | Describe methods used for assessing risk of bias of individual studies (including specification of whether this was done at the study or outcome level), and how this information is to be used in any data synthesis. | 5, 6 |
| Summary measures | 13 | State the principal summary measures (e.g., risk ratio, difference in means). | 6 |
| Synthesis of results | 14 | Describe the methods of handling data and combining results of studies, if done, including measures of consistency (e.g., I^2^) for each meta-analysis. | 6 |

| **Section/topic** | **#** | **Checklist item** | **Reported on page #** |
| --- | --- | --- | --- |
| Risk of bias across studies | 15 | Specify any assessment of risk of bias that may affect the cumulative evidence (e.g., publication bias, selective reporting within studies). | 5, 6 |
| Additional analyses | 16 | Describe methods of additional analyses (e.g., sensitivity or subgroup analyses, meta-regression), if done, indicating which were pre-specified. | - |
| **RESULTS** | | |  |
| Study selection | 17 | Give numbers of studies screened, assessed for eligibility, and included in the review, with reasons for exclusions at each stage, ideally with a flow diagram. | 6, 7, Figure 1 |
| Study characteristics | 18 | For each study, present characteristics for which data were extracted (e.g., study size, PICOS, follow-up period) and provide the citations. | 7, Tables 1 and 2 |
| Risk of bias within studies | 19 | Present data on risk of bias of each study and, if available, any outcome level assessment (see item 12). | 9, Supplementary Tables 3 and 4 |
| Results of individual studies | 20 | For all outcomes considered (benefits or harms), present, for each study: (a) simple summary data for each intervention group (b) effect estimates and confidence intervals, ideally with a forest plot. | 7-10, Tables 1 and 2 |
| Synthesis of results | 21 | Present results of each meta-analysis done, including confidence intervals and measures of consistency. | 10, 11 |
| Risk of bias across studies | 22 | Present results of any assessment of risk of bias across studies (see Item 15). | 10 |
| Additional analysis | 23 | Give results of additional analyses, if done (e.g., sensitivity or subgroup analyses, meta-regression [see Item 16]). | - |
| **DISCUSSION** | | |  |
| Summary of evidence | 24 | Summarize the main findings including the strength of evidence for each main outcome; consider their relevance to key groups (e.g., healthcare providers, users, and policy makers). | 11-14 |
| Limitations | 25 | Discuss limitations at study and outcome level (e.g., risk of bias), and at review-level (e.g., incomplete retrieval of identified research, reporting bias). | 14 |
| Conclusions | 26 | Provide a general interpretation of the results in the context of other evidence, and implications for future research. | 14 |
| **FUNDING** | | |  |
| Funding | 27 | Describe sources of funding for the systematic review and other support (e.g., supply of data); role of funders for the systematic review. | 15 |

*From:*  Moher D, Liberati A, Tetzlaff J, Altman DG, The PRISMA Group (2009). Preferred Reporting Items for Systematic Reviews and Meta-Analyses: The PRISMA Statement. PLoS Med 6(7): e1000097. doi:10.1371/journal.pmed1000097

For more information, visit: **www.prisma-statement.org**.

**Supplementary Table 2.** Method of the database search strategy using PubMed, Scopus, and Web of Science

| **Database (Search**  **conducted up to**  **December, 2024)** | **Search terms^a^** | **Number of studies searched** |
| --- | --- | --- |
| PubMed | ("Chamomile "[Title/Abstract] OR " Camomile "[Title/Abstract]) AND ("PCOS "[Title/Abstract] OR " Polycystic Ovary Syndrome "[Title/Abstract] OR " Polycystic Ovarian Syndrome "[Title/Abstract]) | 8 |
| SCOPUS | (TITLE-ABS-KEY (Chamomile) OR TITLE-ABS-KEY ( Camomile ) AND TITLE-ABS-KEY ( PCOS ) OR TITLE-ABS-KEY ( "Polycystic Ovary Syndrome" ) OR TITLE-ABS-KEY ( " Polycystic Ovarian Syndrome " ) | 180 |
| Web of Sciences | #1 **"** **Chamomile "** (Topic) or **"Chamomile"** (Topic)  #2 **PCOS** (Topic) or **"Polycystic Ovary Syndrome"**(Topic) or **" Polycystic Ovarian Syndrome "**(Topic)  #3 : #1 AND #2 | 5 |
| Google Scholar | "Chamomile" OR "Camomile" AND "PCOS" OR "Polycystic Ovary Syndrome" OR "Polycystic Ovarian Syndrome " | 4 |
| Total |  | 197 |

^a^Searches were limited to original articles, and studies published in the English language using the appropriate filters and/or search terms depending on the database.

**Supplementary Table 3.** Cochrane Collaboration scale for assessment of quality of the included randomized controlled trials (RCTs)

| Study ID | Selection bias | | Performance and detection bias | Attrition bias | Reporting bias | Other bias | The overall risk of bias |
| --- | --- | --- | --- | --- | --- | --- | --- |
|  | **Random sequence generation** | **Allocation concealment** | **Blinding** | **Incomplete outcome data** | **Selective reporting** |  |  |
| Afiat et al., 2024 | Low | Low | Low | Low | Low | Unclear | Low |
| Dadmehr et al., 2023 | Unclear | Low | Low | Low | Low | Low | Low |
| Afiat et al., 2022 | Low | Low | Low | Low | Low | Unclear | Low |
| Heidary et al., 2018 | Low | Low | Low | Low | Low | Unclear | Low |

Low, low risk of bias; Unclear, unclear risk of bias

| **CATEGORY** | **QUESTIONS** | Shamsi et al., 2023 | Alahmadi et al., 2021 | Alahmadi et al., 2020 | Zafari et al., 2010 |
| --- | --- | --- | --- | --- | --- |
| Selection Bias | 1. Was administered dose or exposure level adequately randomized? | ++ | + | + | ++ |
|  | 2. Was allocation to study groups adequately concealed? | + | + | + | + |
| Performance Bias | 3. Were experimental conditions identical across study groups? | ++ | ++ | ++ | ++ |
|  | 4. Were the research personnel and human subjects blinded to the study group during the study? | + | + | + | - |
| Attrition/Exclusion Bias | 5. Were outcome data complete without attrition or exclusion from analysis? | ++ | ++ | ++ | ++ |
| Detection Bias | 6. Can we be confident in the exposure characterization? | ++ | ++ | ++ | ++ |
|  | 7. Can we be confident in the outcome assessment? | ++ | + | + | + |
| Selective Reporting Bias | 8. Were all measured outcomes reported? | ++ | ++ | ++ | + |
| Other Sources of Bias | 9. Were there no other potential threats to internal validity (e.g., statistical methods were appropriate and researchers adhered to the study protocol)? | ++ | ++ | ++ | + |
| Overall Tier | | 1 | 1 | 1 | 2 |
| 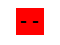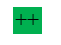Risk of bias response options for individual items:  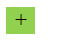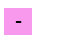 Definitely low risk of bias Definitely high risk of bias Probably low risk of bias Probably high risk of bias | | | | | |

**Supplementary Table 4:** The risk of bias assessment and tier classifications of animal studies (OHAT tool)
